# Incidence of and socio-biologic risk factors for spontaneous preterm birth in HIV positive Nigerian women

**DOI:** 10.1186/1471-2393-12-93

**Published:** 2012-09-09

**Authors:** Oliver C Ezechi, Agatha N David, Chidinma V Gab-Okafor, Harry Ohwodo, David A Oladele, Olufunto O Kalejaiye, Paschal M Ezeobi, Titilola A Gbajabiamila, Rosemary A Adu, Bamidele Oke, Zaidat A Musa, Sabdat O Ekama, Oluwafunke Ilesanmi, Olutosin Odubela, Esther O Somefun, Ebiere C Herbertson, Dan I Onwujekwe, Innocent AO Ujah

**Affiliations:** 1 HIV Treatment Centre, Clinical Sciences Division, Nigerian Institute of Medical Research, 6 Edmund Crescent, Yaba, Lagos, Nigeria

**Keywords:** Spontaneous preterm birth/delivery HIV, Pregnancy, Viral load, CD4 count, Low birth weight

## Abstract

**Background:**

Recent studies have identified HIV as a leading contributor to preterm delivery and its associated morbidity and mortality. However little or no information exists in our sub-region on this subject. Identifying the factors associated with preterm delivery in HIV positive women in our country and sub-region will not only prevent mother to child transmission of HIV virus but will also reduce the morbidity and mortality associated with prematurity and low birth weight. This study was designed to determine the incidence and risk factors for preterm delivery in HIV positive Nigerians.

**Method:**

The required data for this retrospective study was extracted from the data base of a cohort study of the outcome of prevention of mother to child transmission at the Nigerian Institute of Medical Research, Lagos. Only data of women that met the eligibility of spontaneous delivery after 20 weeks of gestation were included. Ethical approval was obtained from the Institution’s Ethical Review Board.

**Results:**

181 women out of the 1626 eligible for inclusion into the study had spontaneous preterm delivery (11.1%). The mean birth weight was 3.1 ± 0.4 kg, with 10.3% having LBW. Spontaneous preterm delivery was found to be significantly associated with unmarried status (cOR: 1.7;1.52-2.57), baseline CD4 count <200 cells/mm^3^(cOR: 1.8; 1.16-2.99), presence of opportunistic infection at delivery (cOR: 2.2;1.23-3.57), multiple pregnancy (cOR 10.4; 4.24 – 26.17), use of PI based triple ARV therapy (eOR 10.2; 5.52 – 18.8) in the first trimester (cOR 2.5; 1.77 – 3.52) on univariate analysis. However after multivariate analysis controlling for potential confounding variables including low birth weight, only multiple pregnancy (aOR: 8.6; CI: 6.73 – 12.9), presence of opportunistic infection at delivery (aOR: 1.9; CI: 1.1 – 5.7), and 1st trimester exposure to PI based triple therapy (aOR: 5.4; CI: 3.4 – 7.8) retained their significant association with preterm delivery.

**Conclusion:**

The spontaneous preterm delivery rate among our cohort was 11.1%. HIV positive women with multiple pregnancies, symptomatic HIV infection at delivery and first trimester fetal exposure to PI based triple therapy were found to be at risk of spontaneous preterm delivery. Early booking and non-use of PI based triple therapy in the first trimester will significantly reduce the risk of preterm delivery.

## Background

An estimated 13 million babies are born before 37 completed weeks of gestation annually. Preterm birth is the single most important cause of neonatal deaths and disability and directly accounts for a quarter of the global neonatal deaths. South central Asia and Sub-Saharan African countries are disproportionately affected by preterm birth and carry a greater burden of death and disabilities attributed to preterm birth [1-4].

As achieving MDG 4 is dependent on high coverage of evidence- based interventions to prevent preterm delivery and to improve survival for preterm newborns, further research efforts are urgently needed to better understand context-specific pathways leading to preterm birth [2,5,6]. This is especially so in HIV high burden countries like Nigeria where, though many interventions have been evaluated, only very few have moderate to high quality evidence for decreasing preterm birth [2,6-8].

Nigeria with 3.6% HIV prevalence and burden of 3.3 million contributes significantly to the global HIV burden. With women accounting for about 60% of the total HIV burden, the coexistence of HIV and pregnancy is thus a common finding. The 2010 national surveillance of HIV infection among pregnant women reported a prevalence of 4.1% and estimated 210,000 HIV positive pregnant women [9-11]. With annual birth of 56,000 HIV infected babies, Nigeria contributes approximately 10% of the global mother to child transmission of HIV infection [9,11]. Reducing this burden by the global target of 90% by 2015 requires the prevention of the causes and factors associated with increased mother to child transmission of HIV infection.

Preterm delivery is one of the factors that have been identified as a major risk factor for mother to child transmission of HIV infection [12-15]. Studies have also identified HIV as a major cause of spontaneous preterm delivery [8,12-15]. Identifying the factors associated with spontaneous preterm delivery in HIV positive women will not only prevent MTCT but will also reduce the morbidity and mortality associated with prematurity and low birth weight. This study was designed to determine the incidence and risk factors for spontaneous preterm delivery in HIV positive Nigerian women, with the aim of using the information obtained to plan prevention strategies.

## Method

### Study setting

The study was conducted at the HIV treatment centre, Nigerian Institute of Medical Research, Lagos. The centre started operation in 2002 following the commencement of the Federal Government of Nigeria ARV access programme. It was included in the 25 centres across the country to give research back up to the national ARV access programme. In 2004 it became one of the centers supported by the Harvard School of Public Health (HSPH), Boston through the PEPFAR Fund. The centre currently provides comprehensive, HIV care, treatment and support for 16, 679 patients (64.6% are women). Sixty five percent of the patients come from Lagos and the rest from the other 5 states of southwestern Nigeria as well as from north-central, south-south and south-eastern Nigeria. A little over 0.025% comes from the neigbouring West African countries. No user fee is charged at the centre. Patients are enrolled into the HIV treatment programme following a referral from the HIV Counseling and Testing Centre, Nigerian Institute of Medical Research Lagos or transfer from other government of Nigeria HIV treatment centres.

HIV positive pregnant women are seen and registered for PMTCT services at the Wednesday PMTCT clinic. While the antenatal and postnatal services, including infant post exposure prophylaxis are provided by the centre, the intrapartum care is provided in collaboration with Lagos University Teaching Hospital, Idi Araba; Lagos State University Teaching Hospital, Ikeja; General Hospitals Surulere, Apapa, Ikorodu; Havana Specialist Hospital Surulere; Rao Specialist Hospital Surulere, and a number of Catholic mission and private hospitals. Health workers from these centres have been trained on intrapartum care of HIV positive mothers either by our centre, the state or national HIV programme. The mothers are referred to any of the centres nearest to their place of residence at 36 weeks or as soon as possible with detailed information about their chosen mode of delivery, infant feeding choice, and Viral Load and CD4 count results. Infant post exposure drug and mothers ARV drugs are also given to the women. After their delivery at the facility, the women are referred back to our centre at 2 weeks post-delivery with a completed Case Record Form (CRF) designed specifically to capture all delivery related information. Information on the CRF is used to complete the postnatal data base. The home based care team contacts any mother who does not report back to centre at 2 weeks post expected date of delivery to ascertain the reasons for the default.

### Study population

All HIV positive women enrolled into our PMTCT programme between July 2004 and June 2010 that met the eligibility criteria were included into the study. Eligibility criteria for this study was based on the WHO definition of viability, that is a birth weight of ≥ 500 g and born at ≥ 20 weeks of gestation. Gestational age was estimated by the number of days between the first day of the last menstrual period (LMP) and date of birth expressed in completed weeks after LMP. A preterm birth was defined as births of infants occurring at less than 37 completed weeks of gestation. Cases of preterm births as a result of medical/therapeutic indications were excluded. Births without information on vital status, birth weight and gestational age were excluded.

### Antiretroviral regimen used during the study period

Antiretroviral drug regimen used during the study period changed over time as a result of the change in national PMTCT guidelines. The regimen changed thrice between July 2004 and June 2010. From July 2004 till March 2006, because of non-availability of widely accepted Nigerian national PMTCT guideline, HAART based regimen was used for PMTCT except for women who presented in labour who were given single dose Nevirapine with Combivir (Zidovudine and Lamivudine) tail of seven days. Between March 2006 and December 2009, the then national PMTCT guideline was nationally adopted and our centre switched to the dictates of the guideline. Triple ARV therapy (HAART) was used only for women eligible for it based on the clinical stage of their HIV disease or CD4 count less than 200 cell/mm3. Those with CD4 count above 200cells/mm3 were placed on ART prophylaxis of either monotherapy (Zidovudine) or dual therapy (Zidovudine + Lamivudine) depending on the gestational age at booking. From January 2010 we reverted back to triple ARV based regimen for all women as the national guideline was revised in line with WHO recommendation. While NNRTI based HAART was given to women with CD4 counts less than 350 cells/mm3, PI based HAART was prescribed for women with CD4 cell count of ≥ 350 cells/mm3.

### Data management

Information on all HIV positive pregnant women who completed PMTCT services during the study period and met the eligibility criteria was extracted from the centres PMTCT Data base which was collected prospectively. For each birth we extracted information on the date of birth, booking status, maternal age and parity, height and weight, marital status, previous obstetric history, estimated gestational age, birth weight, sex and vital status of the baby at birth and whether the infant was a single or multiple delivery. Information on CD4 count, viral load, opportunistic infection status at delivery, type of ARV drug regimen, time of intiation of triple ARV and duration was also collected.

A mother was considered as ‘booked’ if both her pregnancy and HIV status were assessed; laboratory results reviewed and decision taken on the management of her pregnancy. Social status classification by Olusanya and colleagues [16] which was validated for use for the classification of social status of Nigerian women was used. In this system women were classified to belong to one of 5 social classes based on a combination of their educational status and the occupation of their spouse (Class I – V). While classes I and II denote upper social class, classes III and IV, and class V represent middle and lower social classes respectively.

A total of 1843 pregnant HIV positive women booked for PMTCT services during the period with 96.2% (1789) completing the PMTCT services. A total of 1812 babies were delivered by the 1789 women, comprising of 1766 singleton and 23 twin deliveries. One hundred and sixty three cases were excluded from the 1789 (9.3%) births because gestational ages were less than 20 weeks (11.0%), information on birth weight, and gestational age was missing because they delivered outside a hospital facility (34.4%), no vital status information (16.6%), baby weighed less than 500 g despite the LMP date given by the women (14.4%), did not give consent for their data to be uploaded (1.8%), viral load and CD4 results could not be traced (19.0%) and estimated gestational age above 46 weeks (2.5%) but birth weights less than 2.5 kg.

Frequency distributions were generated and univariate analysis using relevant statistics was performed to identify factors associated with spontaneous preterm delivery. Multivariate logistic regression was used to identify independent risk factors for preterm births while controlling for potential confounders including low birth weight, stage of HIV disease, reproductive tract infection and medical disorders. Variables were entered into the model if their P value on univariate analysis was 0.25 or less. The variable with the strongest association in the univariate model was estimated first, followed by others in descending order. In the analysis, the comparison group was term deliveries (a birth at 37–42 completed weeks of gestation). P < 0.05 was considered to be statistically significant. Odds Ratios (OR) and 95% Confidence Intervals (CI) for the OR were also calculated.

### Ethical issues

Approval for the study was obtained from the Institutional Review Board, Nigerian Institute of Medical Research, Lagos Nigeria. Written informed consent was obtained from all women for the use of their data for study. However women who declined consent to participate in the study were provided care but excluded from research. The clinic patients are organized into an independent support group of people living with HIV (Positive Life Organization of Nigeria) that ensures that patients are not stigmatized and discriminated against. This group ensures that no patient is denied requisite care because of failure to participate in any of our studies including this study.

## Result

One thousand six hundred and twenty six women completed the PMTCT programme and fulfilled the eligibility for inclusion into the study. A total 1649 babies were delivered by the 1626 women; 46 of whom were products of twin gestation. The gestational age at booking ranged from 6 to 40 weeks with a mean of 28.6 ± 8.7 weeks. While three hundred and twenty six (20.1%) women registered for PMTCT services during the first trimester, 509 (31.3%) and 791(48.6%) registered during the second and third trimesters respectively.

Eight hundred and forty seven (52.1%) of 1626 women received HAART based regimen (Triple ARV therapy), out of which only 3.5% (57) were on PI based HAART regimen. Three hundred and nine (36.5%) of the 847 women on triple ARV therapy received HAART in the 1st trimester because they were either on it before pregnancy (212; 68.6%), or started on it for their own disease or for PMTCT prophylaxis (97; 31.4%). The remaining 538(63.5%) women on triple regimen were commenced on it during second (288; 34.0%) and third (250; 29.5%) trimesters respectively. In all 635 (74.9%) of the women that received triple ARV therapy were started on it after booking. Of the 779 (47.9%) women that received non triple ARV regimen (single or dual therapy), majority were on Lamivudine + Zidovudine combination (63.7%) and the remaining women received either Zidovudine only regimen (29.4%) or single dose nevirapine in labour (7.9%).

One hundred and eighty one of the eligible 1626 women delivered before 37 completed weeks; a spontaneous preterm delivery rate of 11.1% (95% CI: 8.31- 19.6).

Table 1 shows the relationship between the women’s sociodemographic characteristics and preterm labour. Significant association were only found between women who were not currently married (cOR: 1.7; 95 CI: 1.12-2.57), or engaged in stressful work (cOR :1.4; CI: 1.01-2.06) and preterm delivery.

**Table 1:** Sociodemographic characteristics of the HIV positive pregnant women (n = 1626)

**Factors**	**Preterm Cases (%)**	**No Preterm (%)**	**P value**	**OR**	**95% CI**
	**N=181**	**N=1445**			
**Age (years)**					
· <20	7 (3.9)	43 (3.0)	0.63	1.3	0.54 – 3.57
· 20 – 35	151 (83.4)	1243 (86.0)		1.0	1.0
· >35	23 (12.7)	159 (11.0)	0.55	1.2	0.72 – 1.94
**Parity**					
· 0	54 (29.8)	316 (21.9)	0.20	1.3	0.87 – 1.94
· 1 – 2	71 (39.3)	541 (37.4)		1.0	1.0
· >2	56 (30.9)	588 (40.7)	0.10	0.7	0.49 – 1.07
**Marital status**					
· Married	145 (80.1)	1261 (87.3)		1.0	1.0
· Not married	36 (19.9)	184 (12.7)	<0.05	1.7	1.12 – 2.57
**Work status**					
· **Working**	138 (76.2)	1068 (73.9)	0.59	1.12	0.77–1.64
· **Not working**	43 (23.8)	374 (26.1)		1.0	1.0
**Stress at work**					
· Stressful	90 (65.2)	614 (57.3)	0.04	1.4	1.01 – 2.06
· Not stressful	48 (34.8)	457 (42.7)		1.0	1.0
**Social Class**[16]					
· I & II	20 (11.0)	146 (10.1)	0.70	0.70	0.48 – 1.56
· III	49 (27.1)	415 (28.7)		1.0	1.0
· IV & V	112 (61.9)	884 (61.2)	0.87	0.9	0.54 – 1.59

The effect of biological and anthropometric factors of CD4 count, viral load, BMI, presence of opportunistic and reproductive tract infections at delivery on preterm delivery is summarized in Table 2. There was significant association between all the factors and preterm delivery, except for HIV viral load which showed no association with preterm delivery.

**Table 2:** Biological and anthropometric characteristics of the HIV positive pregnant women (n = 1626)

**Factors**	**Preterm Cases (%)**	**No Preterm (%)**	**P value**	**OR**	**95% CI**
	**N=181**	**N=1445**			
**CD4 level**					
· <200	47 (26.0)	265 (18.3)	0.008	1.8	1.16 – 2.99
· 200 – 350	49 (27.1)	410 (28.4)	0.36	1.3	0.79 – 2.00
· 351–500	40 (22.0)	421 (29.1)		1.0	1.0
· >500	45 (24.9)	349 (24.2)	0.22	1.4	0.83 – 2.18
**Viral load**					
· <1000	79 (43.6)	741 (51.3)		1.0	1.0
· 1000 – 10,000	48 (26.5)	322 (22.2)	0.10	1.4	0.94 – 2.08
· >10,000	54 (29.9)	382 (26.4)	0.16	1.3	0.90 – 1.94
**Body Mass Index**					
· <18.50	12 (6.7)	101 (7.0)	0.94	0.96	0.46 – 1.83
· 18.50 - 24.99	98 (54.1)	791 (54.8)		1.0	1.0
· > = 25	71 (39.2)	553 (38.2)	0.89	1.04	0.74 – 1.45
**Opportunistic Infection at delivery**					
· Present	21 (11.6)	85 (5.9)	0.01	2.1	1.23 – 3.57
· Absent	160 (88.4)	1360 (94.1)		1.0	1.0
Reproductive tract infection at delivery					
Present	13 (7.2)	51 (3.5)	0.03	2.1	1.07–4.11
Absent	168 (92.8)	1394 (96.5)		1.0	1.0

Table 3 shows the relationship between the women’s past obstetric and gynaecological history and preterm delivery. None of the variables showed significant association with preterm delivery.

**Table 3:** Past Obstetric and Gynaecological characteristics of the HIV positive pregnant women (n = 1626)

**Factors**	**Preterm Cases (%)**	**No Preterm (%)**	**P value**	**OR**	**95% CI**
	**N=181**	**N=1445**			
**Induced abortion history**					
· 0	61 (33.0)	566 (39.0)		1.0	1.0
· 1	64 (35.0)	476 (32.0)	0.28	1.3	0.85 – 1.84
· ≥ 2	56 (30.0)	403 (27.0)	0.23	1.3	0.86 – 1.93
**Spontaneous abortion**					
· 0	145 (80.1)	1175 (81.3)		1.0	1.0
· 1	27 (14.9)	198 (10.3)	0.7	1.1	0.70 – 1.74
· ≥ 2	9 (5.7)	92 (4.9)	0.6	0.8	0.37 – 1.66
**Preterm delivery**					
· Yes	9 (5.0)	41 (2.8)	0.8	1.8	0.80 – 3.91
· No	172 (95.0)	1404 (97.2 )		1.0	1.0
**Operative vaginal delivery**					
· Yes	11 (6.1)	56 (3.9)	0.2	1.0	1.0
· No	170 (97)	1389 (96.1)		1.6	0.78 –3.24

Table 4 shows the association between current obstetric and HIV treatment history of the women and preterm delivery. Multiple pregnancy (cOR 10.4; 4.24 – 26.7), use of PI based regimen (cOR 10.2; CI: 5.52-18.8), initiation of triple regimen in the first trimester (cOR:2.5;CI:1.77-3.52) and presence of OIs at delivery (cOR 2.6; 1.63 – 4.52) were found to be significantly associated with preterm delivery . Medical disorders were not found to be associated with preterm delivery in this cohort (cOR:0.97; CI: 0.44-2.04).

**Table 4:** Current Obstetric and HIV treatment characteristics of the HIV positive pregnant women (n = 1626)

**Factors**	**Preterm Cases (%)**	**No Preterm (%)**	**P value**	**OR**	**95% CI**
	**N=181**	**N=1445**			
**Bleeding in pregnancy**					
· Yes	17 (9.4)	137 (2.6)	0.92	1.1	0.60 – 1.85
· No	164 (90.6)	1408 (97.4)		1.0	1.0
**Sex of baby**					
· Male	81 (44.8)	752 (51.7)	0.06	0.7	0.53 – 1.01
· Female	103 (55.2)	703 (48.3)		1.0	1.0
**Multiple pregnancy**					
· Yes	13 (7.1)	10 (0.7)	0.00	10.4	4.24 – 26.17
· No	178 (92.9)	1435 (99.3)		1.0	1.0
**ARV Regimen used for PMTCT**					
· Non PI based HAART Regimen	80 (44.2)	710 (49.1)	0.61	1.11	0.78–1.57
· PI Based HAART Regimen	29 (16.0)	28 (1.9)	0.000	10.2	5.52–18.8
· Non HAART Regimen	72 (39.8)	707 (48.9)		1.0	1.0
**Duration of ARV drugs**					
· **<**8weeks	47 (32.4)	307 (27.1)	0.42	1.2	0.79 – 1.82
· 8-13 weeks	67 (46.2)	525 (46.4)	0.42	0.8	0.51 – 1.30
· >13 weeks	31 (21.4)	299 (26.5)		1.0	1.0
**Period HAART commenced**					
· 1st trimester	96 (53.2)	230 (31.2)	0.00	2.5	1.77 – 3.52
· After 1st trimester	85 (46.8)	508 (68.8)		1.0	1.0
**Medical disorders**					
· Present	9 (5.0)	74 (5.1)	0.92	0.97	0.44 – 2.04
· Absent	172 (95.0)	1371 (94.9)		1.0	1.0

After adjustment for potential confounding variables in multivariate logistic regression including but not limited to controlling for low birth weight, stage of HIV disease, reproductive tract infection and medical disorders, spontaneous preterm delivery in HIV positive women were found to be associated with stressful work (aOR 1.2; CI:1.05- 4.8), OIs (aOR: 1.9; CI: 1.1 – 5.7), multiple pregnancy (aOR: 8.6; CI: 6.73 – 12.9) and 1st trimester use of PI based regimen (aOR: 5.4;CI: 3.4-7.8).

## Discussion

While several studies elsewhere have reported on the prevalence and risk factors for preterm delivery and low birth weight in HIV positive pregnant women, no information on the subject currently exists from our sub region [6-11]. The closest information are reports from the central African countries of Cameroun and Rwanda [6-8]. In addition, the sample size for the two studies from Cameroun and Rwanda were rather small making generalization to Nigeria with the 2nd highest global HIV burden difficult. In this study we determined the incidence and risk factors of preterm delivery using information extracted from prospectively collected data of 1626 HIV positive pregnant women over a 6-year period. Information obtained will assist us and others within our sub region or elsewhere to plan strategies to control and prevent preterm delivery and its associated morbidity and mortality in HIV positive pregnant women.

The preterm delivery rate of 11.1% among the HIV positive pregnant women in this study was similar to 13.5% and 13.6% reported by Towsend and colleagues [11] from United Kingdom and Taguebue and colleagues [6] from Cameroun but much lower than 23.5% reported by Dreyfuss and colleagues from Tanzania, East Africa [17]^.^ The relatively low rate of the preterm delivery in our cohort may be related to the fact that most of our patients were diagnosed early as part of routine voluntary HIV testing during antenatal care, allowing for early initiation of treatment of opportunistic infection which could trigger preterm contractions. In addition a significant number was already on treatment before present pregnancy and only a small proportion of them were on PI based HAART(3.5%) which has been shown to be associated with preterm delivery. The cohorts from Tanzania had a high rate of nutritional deficiency and were in more advanced stages of HIV disease compared to our cohort [6] and thus a high rate of over 23%.

The preterm rate was however significantly higher than rates of 5.8% and 8.5% reported among Nigeria women of unknown HIV status by Etuk [6] and Chike Obi [7] from Nigeria . This higher rate may be attributable to HIV, confirming the findings of the association between HIV and preterm delivery.

In previous studies several factors were identified as risk factors for the development of preterm delivery in HIV positive pregnant women [6-11], However only multiple pregnancy, stressful work, presence of opportunistic infection at delivery and first trimester exposure to PI based triple ARV therapy were found to be associated with preterm delivery in this cohort. Our study utilized a larger sample size and methodologically collected data and thus may have been able to remove the background noise that may have influenced the result of other studies. In addition those studies were from regions with different HIV epidemiology. While the HIV incidence is in single digits in west Africa, in east Africa it is in double digits and the epidemic there is more matured. The viral subtypes and genotypes are also quite different.

The most important predictor of preterm delivery in this cohort was multiple pregnancies (aOR: 8.6; CI: 6.73 – 12.9). Though this finding is similar to findings of Taguebue and colleagues from central Africa [6], studies from UK [11] and East Africa [9] did not identify multiple pregnancies as contributing to preterm delivery in HIV positive women. It is also important to note that in our earlier study among HIV negative population from the same region of Nigeria where the current study was conducted, we identified multiple pregnancies as a major risk factor for preterm delivery [5]. Our study thus showed that irrespective of HIV status, multiple pregnancies remains a significant cause of preterm delivery in our country. Women with multiple pregnancies tend to develop preterm delivery because of uterine over distention (malpresentation, polyhydramionos), and premature rupture of membrane, antepartum haemorrhage and intrauterine growth retardation [1,4,5]^,^ which may trigger uterine contractions.

Opportunistic infections at delivery (aOR: 1.9; CI: 1.1 – 5.7), stressful work (aOR 1.2; CI: 1.05- 4.8) and first trimester exposure to PI based HAART (aOR: 2.4; CI: 1.07 – 3.15) were the other factors shown to be associated with preterm delivery in our cohort. While the association of the presence of OIs and stressful work with preterm delivery in HIV positive women seems obvious, that of first trimester PI based HAART exposure was not immediately obvious. However it is in keeping with previous reports which showed that PI based HAART is associated with preterm delivery [11]. It is also important to state that while other studies were not able to associate the period of HAART and preterm delivery we were able to show that only first trimester PI based HAART exposure and not Non PI based HAART or its duration was associated with preterm delivery. There has been ongoing debate on the role of PI based regimen on preterm delivery rate [18-26]. While some studies reported an association between PI use and preterm delivery [20-23], others have reported no association [24,25] or have been underpowered to detect a modest increase in preterm deliveries [26]. The results of this study supports other studies that linked preterm delivery to use of PI based regimen [20-24]. In our study we not only used a large sample size but also controlled for possible confounders. Our study was also sufficiently powered to detect a two times risk of developing preterm delivery in pregnant HIV positive women who were exposed to first trimester PI based HAART.

The presence of reproductive tract infection at delivery which was found to be associated with preterm birth at univariate analysis did not retain its significant association with preterm delivery after controlling for the effect of co-morbidity of opportunistic infection. In addition the small proportion of women with reproductive tract infection at delivery in this cohort may have contributed to loss of significance. As a result of the great aversion for caesarean section among Nigerian women [27], we actively screen for and treat vaginal infection before referral at 36 weeks even when the women have been previously treated. The active screening and prompt treatment of reproductive tract infection ensured that only few women still had reproductive tract infection at delivery.

Though our study tried to eliminate the weakness of other previous studies, we had the limitation of its being a retrospective study. However the data used for this analysis, though retrospectively assembled is part of a methodologically collected data base of a PMTCT follow up study in our facility. Data used were extracted from a prospectively collected data using case record form designed for the PMTCT follow up study thus ensuring the quality and completeness of data used in this study.

## Conclusion

This study shows that HIV positive Nigerian women are at increased risk of preterm delivery, more especially so in women with multiple gestations, opportunistic infection close to delivery, who engaged in stressful work and commenced on PI based HAARTduring the first trimester.

## Competing interests

The authors declare that they have no competing interests.

## Authors’ contribution

OCE, AND, CVGO: Conceive the study, revised the initial design of the study, participated in the analysis of the data, critically revised draft manuscripts for important intellectual content. HO, DAO, OOK, PME, TAG,BO: Produced the initial design of the study, interpreted the analyzed data, contributed intellectually to the revisions of the manuscript and gave final approval. RAA, ZAM, SOE, OI, OO, EOS, ECH: Participated in the redesign of the initial draft design, data collection and entry, drafted the initial manuscript, incorporated all the suggested changes and produced the final manuscript. DIO and IAOU: Developed the analysis plan, interpreted the analyzed data, revised the manuscripts critically for important intellectual content. All authors read and approved the final manuscript.

## Pre-publication history

The pre-publication history for this paper can be accessed here:

http://www.biomedcentral.com/1471-2393/12/93/prepub
